# Alpha-tocopherol concentration in serum and colostrum of mothers with gestational diabetes mellitus

**DOI:** 10.1590/0103-0582201432214113

**Published:** 2014-06

**Authors:** Fernanda Barros S. Resende, Heleni Aires Clemente, Dalila Fernandes Bezerra, Evellyn Câmara Grilo, Larisse Rayanne M. de Melo, Paula Emília N. R. Bellot, Raquel Costa S. Dantas, Roberto Dimenstein

**Affiliations:** 1 UFRN, Natal, RN, Brasil

**Keywords:** diabetes, gestational, colostrum, serum, alpha-tocopherol

## Abstract

**OBJECTIVE:**

To evaluate and compare the levels of α-tocopherol in colostrum and in the serum of healthy and diabetic mothers.

**METHODS:**

This cross-sectional study enrolled 51 volunteer mothers, 20 with the diagnosis of gestational diabetes mellitus and 31 without associated diseases. Serum and colostrum samples were collected in fasting in the immediate postpartum period and α-tocopherol was analyzed by high performance liquid chromatography (HPLC). In order to define the nutritional status of vitamin E, the cutoff point for the serum (697.7µg/dL) was adopted. Student's t-test for independent variables compared the average concentrations of α-tocopherol in the serum and in the colostrum between control and gestational diabetes mellitus groups. Pearson's correlation was used to assess the relationship between the concentration of α-tocopherol in serum and colostrum for both groups. Differences were considered significant when *p*<0.05.

**RESULTS:**

The α-tocopherol concentration in colostrum was 1,483.1±533.8µg/dL for Control Group and 1,368.8±681.8µg/dL for diabetic women, without differences between groups (*p*=0.50). However, α-tocopherol concentration in the serum was 1,059.5±372.7µg/dL in the Control Group and 1,391.4±531.5µg/dL in the diabetic one (*p*<0.01). No correlation was found between the concentration of α-tocopherol in the serum and in the colostrum for control and diabetic groups.

**CONCLUSIONS:**

The groups had adequate nutritional status of vitamin E. Gestational diabetes was not associated with changes in α-tocopherol concentration in colostrum.

## Abstract (es)

**OBJETIVO:**

Evaluar y comparar la concentración de α-tocoferol en la leche calostro y en el suero de madres diabéticas y sanas.

**MÉTODOS:**

Estudio transversal, realizado con 51 parturientes voluntarias, siendo 20 diagnosticadas con diabetes mellitus gestacional y 31 sin cualquier enfermedad asociada. Se recogieron las muestras de suero y de calostro en ayuno en el postparto inmediato y se analizó el α-tocoferol por cromatografía líquida de alta eficiencia (CLAE). Para definir el estado nutricional de vitamina E, se adoptó el punto de corte sérico (697,7µg/dL). El análisis estadístico se realizó con la prueba *t* de Student para variables independientes a fin de comparar las concentraciones medianas de α-tocoferol en el suero entre los grupos control y con diabetes mellitus gestacional. Ese análisis también fue realizado para comparar los promedios de α-tocoferol en el calostro en los grupos estudiados. Se utilizó la correlación de Pearson para evaluar la relación entre la concentración de α-tocoferol en el suero y en el calostro para ambos grupos. Las diferencias fueron consideradas significativas cuando *p*<0,05.

**RESULTADOS:**

Las concentraciones de α-tocoferol en el calostro fueron 1.483,1±533,8µg/dL para las mujeres del Grupo Control y 1.368,8±681,8µg/dL para las diabéticas, no habiendo diferencias (*p*=0,50). Sin embargo, en el suero de las puérperas control, la concentración de α-tocoferol fue 1.059,5±72,7µg/dL y, en las diabéticas, 1.391,4±531,5µg/dL, con *p*<0,01). No hubo correlación entre la concentración de α-tocoferol en el suero y en el calostro para el Grupo Control. Resultado semejante fue encontrado para el grupo con diabetes mellitus gestacional.

**CONCLUSIONES:**

Los grupos presentaron estado nutricional adecuado respecto a la vitamina E. No hubo asociación entre diabetes mellitus gestacional y concentración de α-tocoferol en el calostro.

## Introduction

During pregnancy, there is usually an accumulation of energy reserves, characterized by common maternal physiological responses to pregnancy, such as increased visceral fat, insulin resistance, and increased circulating lipids^(^ 1 ^)^. Insulin resistance promotes the increase in glucose concentration, characterizing a scenario of gestational diabetes mellitus (GDM). Studies have shown the occurrence of increased production of oxygen free radicals, supporting the hypothesis that there is increased oxidative stress in patients with gestational diabetes^(^ 2 ^)^. However, there are still some knowledge gaps regarding oxidative stress during pregnancy and their reflection on neonates.

GDM carries several mother-baby risk factors, among them, the increased incidence of teratogenicity, higher in fetuses of diabetic mothers when compared to healthy mothers, being this malformations also resulting from the cellular damage caused by the activity of free radicals^(^ 2 ^)^.

The natural antioxidants reduce the adverse effects of free radicals because they have the ability to capture and neutralize reactive oxygen species (ROE), preventing lipid peroxidation. This neutralization is essential, especially in situations of increased oxidative stress, as in GDM^(^ 3 ^)^. Among these substances, vitamin E stands out, essential micronutrient that corresponds to a group of eight fat soluble compounds classified as α-, β-, γ-, and δ-tocopherol or tocotrienol, being α-tocopherol the most active biologically^(^ 4 ^)^. 

It is known that the placental transfer of vitamin E during pregnancy is limited and reservations of this micronutrient formed in the newborn are low, making exclusive breastfeeding the only source of acquisition to meet their nutritional needs^(^ 4 ^)^. This offer is extremely important, since the exposure to hyperoxia at birth increases the risk of formation of free radicals^(^ 5 ^)^. Researchers evaluated the relationship between diabetes and vitamin E and found that its supplementation is related to the production of insulin and the protection of pancreatic beta cells, revealing that vitamin E might be directly related to diabetes^(^ 6 ^)^. 

Given the importance of vitamin E to the mother-child binomial, this study aimed to verify the alpha-tocopherol concentration in serum and colostrum of mothers with gestational diabetes mellitus and in healthy mothers, as well as to compare the vitamin values between groups. The relevance of this work consist on verifying whether GDM can be a risk factor for vitamin E deficiency in parturient women and/or newborns, considering that breast milk is the only source of this vitamin to newborns in exclusive breastfeeding. 

## Method

The present study has a cross-sectional design, and was conducted with 51 volunteer mothers, 20 diabetic and 31 without associated diseases, all treated in a public maternity service in the municipality of Natal, state of Rio Grande do Norte. The samples were collected from October 2011 to August 2012.

For the sample size calculation, we used the G*Power *software*, version 3.1.7^(^ 7 ^)^. The calculation was performed considering the following parameters: α=5%, 80% test power, and expectation of the effect measure of 0.81. The total size indicated for the sample was of 50 cases.

Exclusion criteria for the group of healthy parturient women were: presence of diseases (diabetes, hypertension, neoplasia, gastrointestinal tract, and liver diseases, heart diseases, infectious syphilis, and HIV positive); use of vitamin supplements containing vitamin E during pregnancy; multiple fetuses or with malformations. Regarding mothers with gestational diabetes, the insulin-depended, those with multiple fetuses or fetuses with malformations, and those who used vitamin supplements during pregnancy were excluded. The diagnosis of GDM was made by the medical staff during the prenatal of the participants. For positive screening during prenatal care regardless of the gestational period, it was established that maternal fasting blood glucose should be ≥85mg/dL. For the diagnosis of GDM, we used the cutoffs of 110mg/dL for fasting glucose and of 140mg/dL for the value of the Oral Glucose Tolerance Test (OGTT), performed 2 hours after oral administration of 75g glucose. 

All procedures of the study were approved by the Research Ethics Committee of the University Hospital Onofre Lopes within Universidade Federal do Rio Grande do Norte (UFRN), under protocol n. 325/09. All participants were informed about the study and signed the informed consent. 

Data on maternal and obstetric characteristics were obtained from medical records of the women in labor, the pre-natal care card, and through interviews conducted by the researchers. The pre-pregnancy anthropometric nutritional status was determined by the body mass index (BMI), calculated as the ratio between the normal body weight of the woman before pregnancy and her height squared. Women with BMI <19.8kg/m^2^ were classified as underweight; those with a BMI between 19.8-26kg/m^2^ as eutrophic; women with BMI between 26-29kg/m^2 ^were classified as overweight, and women with BMI>29kg/m^2^ were classified as obese^(^ 8 ^)^. 

The gestational weight gain of participants was calculated as the difference between the pre-partum body weight and the pre-pregnancy weight and assessed in accordance with the recommendations by the Institute of Medicine^(^ 8 ^)^. Women with low pre-pregnancy weight should gain between 12.5-18kg until the end of pregnancy; those with adequate pre-gestational weight, between 11.5-16kg; those with overweight, between 7-11.5kg; and obese women should gain about 7kg until the end of pregnancy.

Data on the characteristics of newborns of participating mothers were obtained from medical records. Newborns were classified according to gestational age in preterm (those born with less than 37 weeks) and term infants (those born between the 37th and the 42th week)^(^ 9 ^)^. The newborns were also classified according to birth weight in underweight (2,500g); adequate weight (weight between 2,500 and 4,000g); and macrosomic (weight above 4,000g)^(^ 10 ^)^. 

On the first day postpartum, 2mL of colostrum and 5mL of blood were collected from each parturient involved in the study. These biological samples were collected in the morning after fasting for about 8 to 12 hours. Colostrum was obtained by manual expression from a single breast to prevent fluctuation in fat, and blood was obtained by venipuncture. The samples were collected in polypropylene tubes, protected from light and transported in coolers to the Laboratory of Food and Nutrition Biochemistry at the Biochemistry Department of UFRN. The colostrum samples were subjected to water-bath at 37°C for 5 minutes and stirred for homogenization of the sample. Then, an aliquot of 500µL was placed in a light-protected polypropylene tube and stored at -18°C until analysis. Blood was centrifuged to remove the serum, and 1mL was separated ad stored with the same procedure performed with the colostrum. 

The alpha-tocopherol concentrations in the samples of serum and colostrum were determined by high performance liquid chromatography (HPLC). Biochemical analysis of α-tocopherol in biological samples occurred according to the adaptation of the extraction method used by Ortega et al^(^ 11 ^)^, as described below. For 1mL of serum, 1mL of ethanol 95% (Merck^(r)^, USA) was used; and then, there were two extractions with 2mL of hexane (Merck^(r)^, USA), and evaporation of 2mL of hexane extract in a water bath at 37ºC. For 500μL of colostrum, 500μL of ethanol 95% (Merck^(r)^, USA) were added; then, two extractions with 2mL of hexane (Merck^(r)^, USA), and evaporation in the same conditions as the serum. After these proceedings, the samples were redissolved in 500μL of absolute ethanol (Vetec^(r)^, Brazil) and 20µL were applied to the HPLC apparatus (model LC-10 AD, Shimadzu Corporation^(r)^, Japan) coupled to a UV-VIS detector (SPD-10 A, Shimadzu Corporation^(r)^, Japan) and to a Chromatopac C-R6A integrator (Shimadzu Corporation^(r)^, Japan). A reverse phase column was used (CLC-ODS (M), Shim-pack, Japan) 4.6mm d.i.x25cm in length. The mobile phase used in these analyses was of methanol 100% (J. T. Baker, Mexico) at a flow rate of 1.0mL/minute. 

The α-tocopherol was identified in the samples by comparing the retention time of the peaks obtained in the chromatograms and that obtained by applying the standard α-tocopherol Sigma^(r)^. In order to quantify α-tocopherol in biological samples, we used a standard curve for external standardization. The concentration of the standard was confirmed by specific extinction coefficient in absolute ethanol^(^ 12 ^)^, ε 1%, 1cm=75.8 to 292nm (Sigma^(r)^, USA). 

Concentrations of maternal α-tocopherol serum levels above 697.7µg/dL are considered acceptable, according to the values established by Sauberlich^(^ 13 ^)^.

To determine the precision and accuracy, there were tests of recovery and repeatability expressed by relative standard deviation (RSD), with three concentrations, (50, 100, and 200ng/mL), contemplating the range of variation within the linearity curve. We also determined the detection limit (DL) and the quantitation limit (QL).

The α-tocopherol was found in colostrum and serum. The average rate of recovery of α-tocopherol in the serum was of 100% and, for colostrum, of 98.8%. The measurements were characterized by satisfactory repeatability, with RSD of serum and colostrum from 7.7 and 4.2%, respectively. 

To determine the DL and the QL, we diluted the samples of serum and colostrum, whose concentrations were known. For each dilution, the samples were applied and the peak was observed, so that the DL was determined when there was no more distinction between the noise and the analytical signal, which was reached in the concentration of 3.18ng/mL. The QL was determined when the analytical signal was detected in the lower dilution, equivalent to 6.36ng/mL, according to the visual method^(^ 14 ^)^. The calibration curve was performed with standard solutions of α-tocopherol (Sigma^(r))^. The calibration curve for α-tocopherol was linear (r^2^=0.9998) and obtained within 1.2-41.3µg/mL. 

We analyzed the data by *Statistic Release *7^(r)^, and they are presented as mean and standard deviation. We used the Kolmogorov-Smirnov test to check the normality of the metric variables of interest. After verification of normality, we used the Student *t* test for independent variables, in order to determine whether there is significant difference between the means of α-tocopherol in the studied groups. We used the Pearson correlation to assess the relationship between the concentration of α-tocopherol in serum and colostrum, establishing significance at *p*<0.05. This correlation was performed for each studied group.

## Results

Parturients who participated in this study were distributed for the analysis of results in two groups: Control (n=31) and gestational diabetes mellitus group (n=20). 

Women included in the control group were mostly adult (81%), were submitted to vaginal delivery (55%), and had term (96%) and normal weight children (87%). As to pre-pregnancy weight, 42% were classified as eutrophic. The mean gestational age (GA) of newborns of parturient women in this group was of 39±1.2 weeks, and only one newborn was classified as pre-term, with a GA of 36 weeks. As for pregnant women included in the diabetic group, all were adults and most had cesareans, (90%) and term children (71%). Regarding the classification of pre-pregnancy weight, 44% were obese. As for the weight of newborns in this group, 33.3% were born underweight, 33.3% had adequate weight, and 33.3% were macrosomic. The mean GA of infants from mothers included in this group was of 37±2 weeks and four infants were classified as preterm, being the mean gestational age of 35±1 weeks (Table 1). 

**Table 1:**
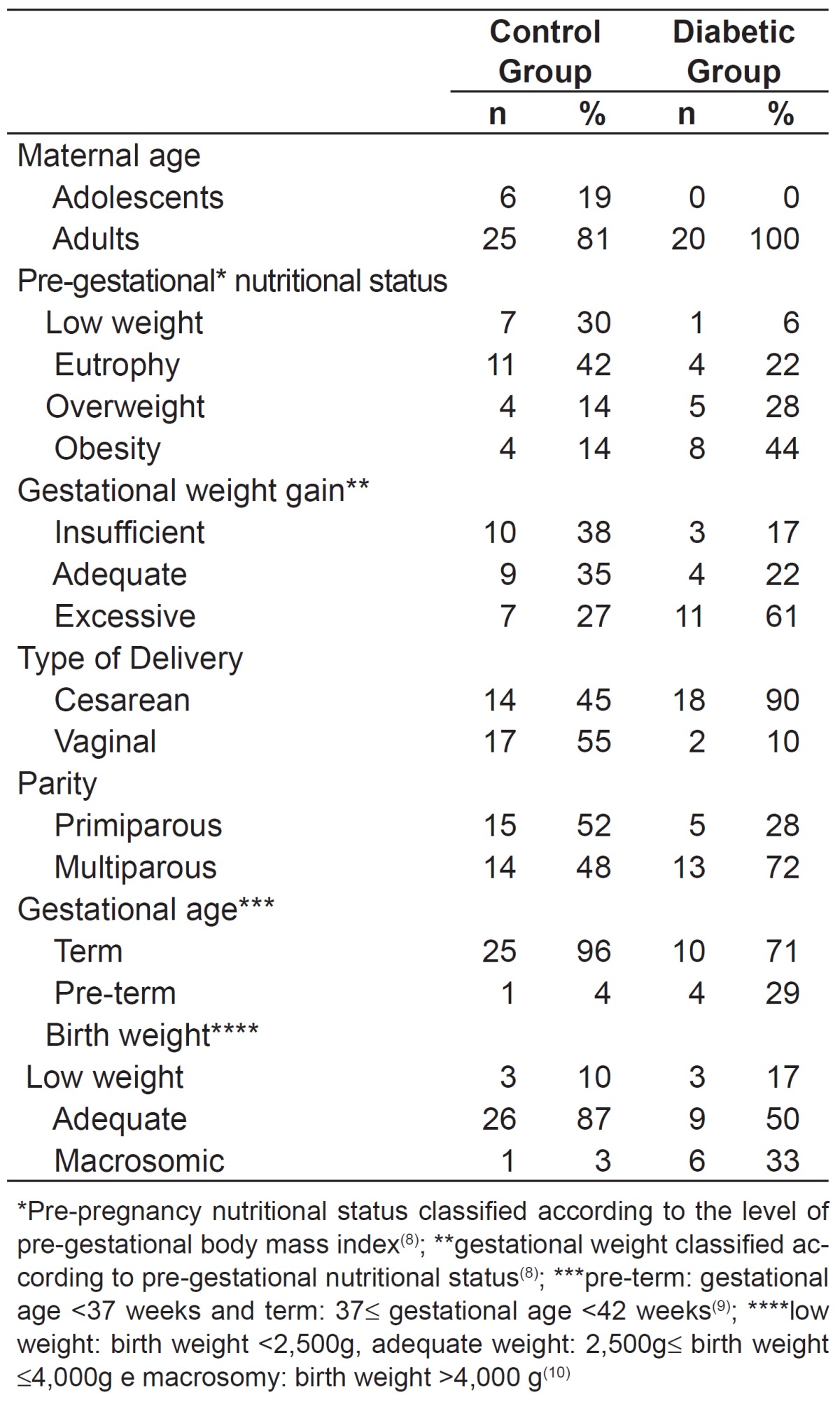
Characteristics of the study population based on maternal, obstetric, and neonate's characteristics

**Figure 1:**
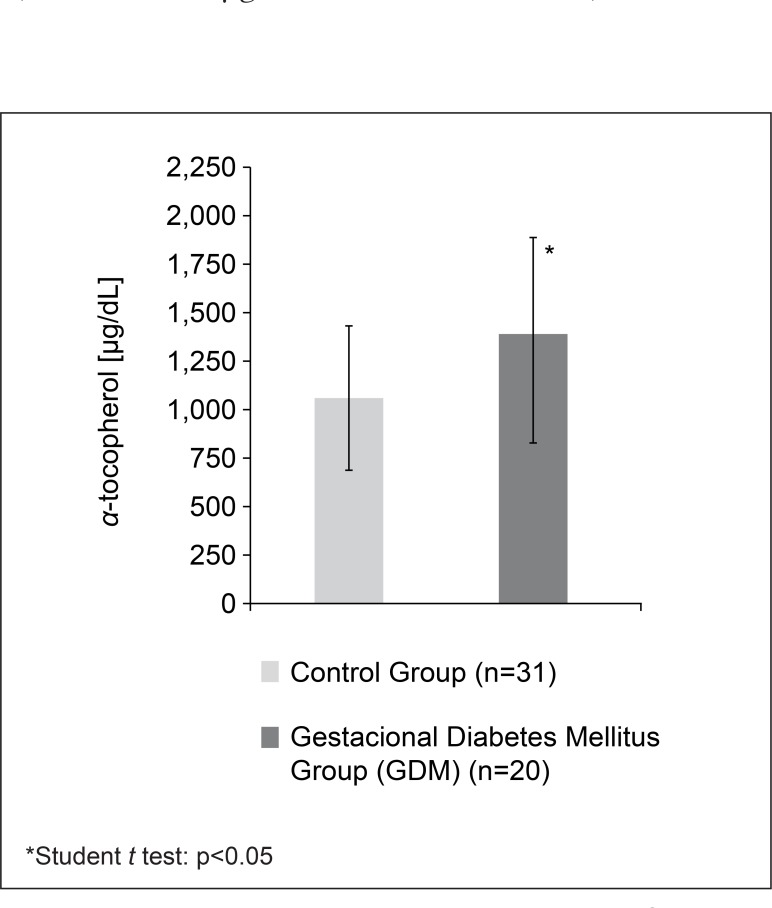
a-tocopherol concentration in serum of lactating women in the Control Group and in the Gestational Diabetes Mellitus Group

**Figure 2:**
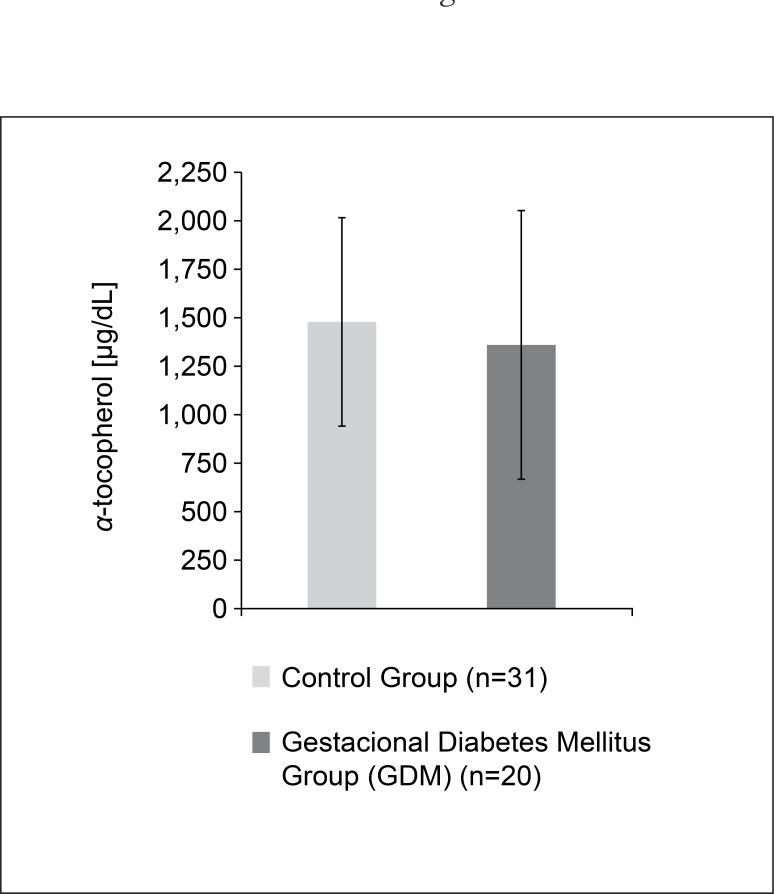
Concentration of a-tocopherol in colostrum of lactating women in the Control Group and in the Group with Gestational Diabetes Mellitus

**Figure 3:**
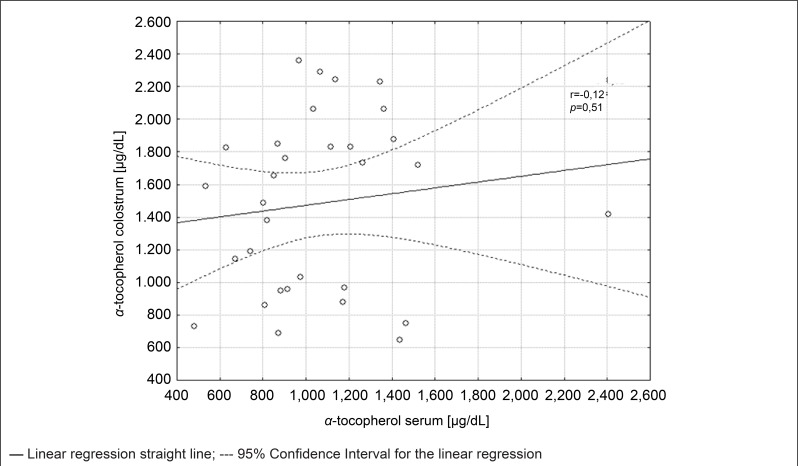
Correlation between a-tocopherol concentration in the serum and colostrum of lactating women in Control Group

**Figure 4:**
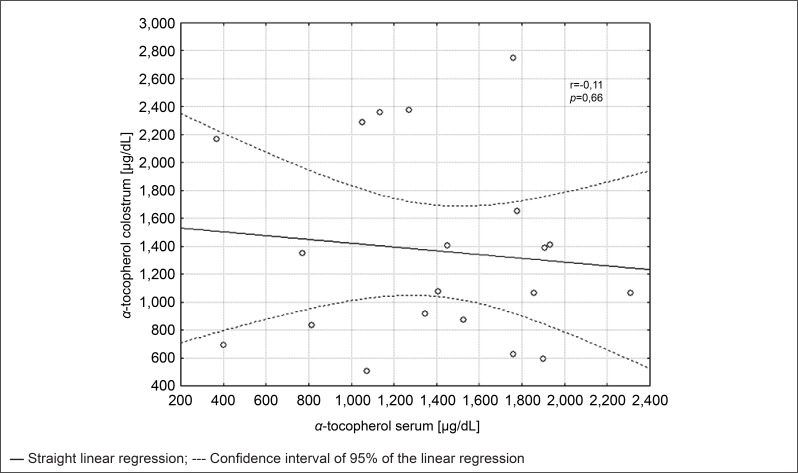
Correlation between the concentration of a-tocopherol in serum and colostrum of lactating women from the Group of Gestational Diabetes Mellitus

In pregnant women in the control group, the mean α-tocopherol concentration found in serum was of 1,059.5±372.7µg/dL (minimum: 482.3; maximum: 2,405.8). For the group of diabetic women, the mean was of 1,391.4±531.5µg/dL (minimum: 368.0; maximum: 2,311.5), with a significant difference between the groups (*p*<0.01) (Figure 1). The α-tocopherol concentration in colostrum was of 1,483.1±533.8µg/dL (minimum: 645.5; maximum: 2,360.1) for participants in Control Group and of 1,368.8±681.8µg/dL (minimum: 505.6; maximum: 2,750.6) for diabetic women, with no difference between groups (Figure 2). No correlation was found between serum and colostrum for both the control group (*p*>0.05; r=0.12) and the GDM group (*p*>0.05; r=-0.11). The correlation for both groups is expressed in Figures 3 and 4. 

## Discussion

The maternal nutritional state pre- and during pregnancy is one of the most important variable factors for the mother-child health. The inadequacy of the maternal nutritional state influences the outcome of pregnancy and the conditions of growth presented by the newborn, as the gestational period is a phase in which the nutritional needs are high, due to physiological adjustments of the pregnant woman and the nutritional demands for fetal growth^(^ 15 ^)^. 

From the means of α-tocopherol serum concentrations observed in both groups, the nutritional biochemical state of the serum is acceptable^(^ 13 ^)^, and there was no deficiency related to diabetes. Regarding the colostrum, it was verified that both groups meet the nutritional requirement of the newborn, considering that the estimated volume of colostrum ingestion is of 500mL/day^(^ 16 ^)^, and the result was compared directly to the 2000 reference value of the Institute of Medicine^(17) ^for the ingestion of the nutrient (4mg/day). 

From the results, it was observed that the serum of women with GDM showed higher α-tocopherol concentrations when compared to healthy mothers. However, Grissa et al^(^ 18 ^)^ and Peuchant et al^(^ 19 ^)^, in a study conducted with pregnant women, reported a decrease in vitamin E concentrations when compared to those of healthy mothers. Suhail et al^(^ 20 ^)^ also found decreased serum concentrations compared to the control group. However, these authors carried out the collection at delivery, unlike this study, in which samples were collected from samples in the first day postpartum. 

In studies conducted by Lammi-Keefe et al^(^ 21 ^)^ and Dey et al^(^ 22 ^)^, there was no significant difference between the amount of tocopherol in the serum of the diabetic group and the healthy group. The study by Lammi-Keefe et al^(^ 21 ^)^ was the first conducted with insulin-dependent diabetes mellitus lactating women and their respective control group, composed of healthy lactating women. The second study was conducted in India and analyzed tocopherol in pregnant women diagnosed with gestational diabetes. However, another study conducted in India by Santra et al^(^ 23 ^)^ found a higher amount of vitamin E in the serum of women with GDM compared to the control group, confirming the results obtained in this research. The amount of vitamin E has undergone gradual increase even after 4 weeks, though new serum collection.

Increased α-tocopherol serum concentration may be related to metabolic changes that occur in the maternal body due to GDM. At the end of pregnancy, there should be a reduction in insulin secretion, leading to a hormonal response that make tissues dependent on insulin to metabolize lipids instead of carbohydrates, initiating a process of lipolysis^(^ 24 ^-^ 26 ^)^, which culminates in increased release of free fatty acids in the circulation^(^ 27 ^)^. Once the primary means of storage of α-tocopherol in the body is the adipose tissue^(^ 28 ^)^, this vitamin can be influenced by increased lipolysis, leading diabetic women to present high concentration of serum tocopherol. 

There is evidence that increased oxidative stress may enhance antioxidant enzyme activity in animals, as an adaptive body condition. De Angelis et al found that diabetic rats had an increase of 92% in catalase activity and of 27% in glutathione S-transferase in muscles, compared to the control group. As vitamin E acts as an antioxidant, it is oxidized and transformed into a free radical (tocopherol), thereby, requiring, a regeneration system which allows the recovery of its antioxidant function. Thus, other antioxidants act removing the free radical from the tocopherol molecule, such as ascorbic acid, reduced-glutathione and Coenzyme Q10^(^ 29 ^,^ 30 ^)^. Giannubilo et al^(^ 31 ^)^ found a significantly higher plasma concentration of coenzyme Q10 in late pregnancy (36-40 weeks) in patients with GDM, when compared to the control group. To explain this difference, the authors suggest that there is a compensatory mechanism in response to oxidative stress associated with hyperglycemia and insulin resistance in patients with GDM. 

Considering the studies analyzed and the results obtained, despite the few published articles, we could hypothesize that the increase in α-tocopherol in the blood of mothers with GDM is related to the attempt to respond to the disease and minimize damages, in the form of compensatory mechanisms observed both in increased lipolysis in the face of low energy availability and in the increase of antioxidant activity against oxidative stress.

As for the concentration of α-tocopherol in colostrum of diabetic mothers, there are few studies on the subject. Despite the scarcity of studies, Lammi-Keefe et al^(^ 21 ^)^ analyzed the amount of vitamin E in the milk of diabetic mothers in the seventh, 14th, 42th, and 84th days of lactation. In their results, it is observed that the amount of tocopherol in colostrum, which corresponds to sampling in the seventh day, was higher compared to the group of healthy mothers, being a significant difference. In the same group, the vitamin concentration was below the reference value adopted by the authors. This same study also found that the α-tocopherol concentrations for diabetic mothers are different from those found in the control group, both in colostrum and in serum; however, in both groups, there was no significant correlation between them, similarly to previous studies, such as those from Azeredo and Trugo^(^ 32 ^)^, Dimenstein et al^(^ 33 ^)^, and Lira et al^(^ 34 ^)^. This reinforces the hypothesis that there is a different mechanism of tocopherol transfer to the mammary gland in order to meet the needs of the newborn, with no dependence on maternal serum status of this nutrient^(^ 32 ^)^.

In this context, the existence of this independent mechanism is essential, because the maternal body must ensure the transfer of vitamin E to milk in sufficient quantities for the formation of the child's reserves, ensuring protection of the organism against oxygen toxicity and stimulating the development and maintenance of the immune system^(^ 4 ^)^. In addition to its action as an antioxidant, which is extremely important to the newborn, vitamin E also acts has a modulating function on the post-translation and transcription of genes, anti-inflammatory action, and in membrane stability^(^ 5 ^,^ 35 ^,^ 36 ^)^.

Both healthy pregnant women and those with GDM presented adequate nutritional status regarding vitamin E. Nevertheless, the concentration of serum α-tocopherol was significantly higher in the diabetic group, being a protective factor against oxidative stress for these women. It is suggested that this increase results from metabolic changes associated with GDM, such as lipolysis.

Regarding study limitations, it is worth mentioning that the test power expresses the probability of detecting a true effect^(^ 37 ^)^. The probability of a researcher committing a type II error, in which he does not discard the null hypothesis when it is false to the population, is designed by beta error (β)^(^ 38 ^)^. For sample calculation of this study, we used an 80% test power and beta error value of 20%. Furthermore, we included some mothers of premature newborns. However, some studies show that gestational age has no influence on the levels of α-tocopherol in colostrum^(^ 39 ^-^ 41 ^)^. Finally, we also mention the fact that no dietary assessment of pregnant women included in the study was performed and the fact that milk collection was performed only in one period of lactation. Nonetheless, works like this, that verify the association between diseases and the concentration of vitamins in serum and breast milk are relevant to define risk groups for vitamin deficiency. 

GDM was not associated with the serum concentration of α-tocopherol in colostrum and there seems to be a risk factor for vitamin E deficiency in newborns whose mothers have this disease. Vitamin concentrations in both groups supply the daily requirement for vitamin E in infants. In addition, mothers with GDM had higher serum concentrations of α-tocopherol, which may be a reflection of physiological and biochemical changes that characterize the disease. Further studies should be conducted to verify the influence of other common diseases in pregnancy on the concentration of α-tocopherol and other vitamins in serum and maternal milk.
