# Supplementary material for: Alpha-tocopherol concentration in serum and colostrum of mothers with gestational diabetes mellitus
Source: Rev Paul Pediatr. 2014 Jun;32(2):178–86. doi: 10.1590/0103-0582201432214113 (PMC4183008; doi:10.1590/0103-0582201432214113)
Supplement: Translation

# Translation: Concentração de α-tocoferol no soro e colostro de mães com diabetes melito gestacional

**Authors:** Fernanda Barros S. Resende, Heleni Aires Clemente, Dalila Fernandes Bezerra, Evellyn Câmara Grilo, Larisse Rayanne M. de Melo, Paula Emília N. R. Bellot, Raquel Costa S. Dantas, Roberto Dimenstein

**Affiliations:** 1 UFRN, Natal, RN, Brasil

**Keywords:** diabetes gestacional, colostro, soro, alfa-tocoferol

## Abstract

**OBJETIVO:**

Avaliar e comparar a concentração de α-tocoferol no leite colostro e no soro de mães diabéticas e saudáveis.

**MÉTODOS:**

Estudo transversal, realizado com 51 parturientes voluntárias, sendo 20 diagnosticadas com diabetes melito gestacional e 31 sem nenhuma doença associada. Coletaram-se as amostras de soro e de colostro em jejum no pós-parto imediato e o α-tocoferol foi analisado por cromatografia líquida de alta eficiência (CLAE). Para definir o estado nutricional de vitamina E, adotou-se ponto de corte sérico (697,7µg/dL). O teste *t* de Student para variáveis independentes comparou as concentrações médias de α-tocoferol no soro e no colostro entre os grupos controle e com diabetes melito gestacional. A correlação de Pearson testou a relação entre a concentração de α-tocoferol no soro e no colostro para ambos os grupos. As diferenças foram consideradas significantes quando *p*<0,05.

**RESULTADOS:**

As concentrações de α-tocoferol no colostro foram 1.483,1±533,8µg/dL para as mulheres do Grupo Controle e 1.368,8±681,8µg/dL para as diabéticas, não havendo diferenças (*p*=0,50). Entretanto, no soro das puérperas controle, a concentração de α-tocoferol foi 1.059,5±372,7µg/dL e, nas diabéticas, 1.391,4±531,5µg/dL, com *p*<0,01. Não houve correlação entre a concentração de α-tocoferol no soro e no colostro para o Grupo Controle. Resultado semelhante foi encontrado para o grupo com diabetes melito gestacional.

**CONCLUSÕES:**

Os grupos apresentaram estado nutricional adequado quanto à vitamina E. Não houve associação entre diabetes melito gestacional e mudanças na concentração de α-tocoferol no colostro.

## Introdução

Durante a gestação, geralmente ocorre um acúmulo de reserva energética, caracterizado por respostas fisiológicas maternas comuns à gestação como aumento da gordura visceral, resistência à insulina e elevação dos lipídios circulantes^(^ ^1^ ^)^. A resistência à insulina promove a elevação na concentração de glicose, caracterizando um quadro de diabetes melito gestacional (DMG). Estudos têm demonstrado a ocorrência de produção maior de radicais livres de oxigênio, apoiando a hipótese de que há um aumento do estresse oxidativo em pacientes com diabetes gestacional^(^ ^2^ ^)^. No entanto, ainda há lacunas sobre o estresse oxidativo na gravidez e seus reflexos sobre o neonato.

A DMG acarreta diversos fatores de risco para o binômio mãe-filho, entre eles, o aumento da incidência de teratogenia, maior nos fetos de mães diabéticas quando comparados aos de mães saudáveis, sendo essas malformações decorrentes também do dano celular causado pela atividade de radicais livres^(^ ^2^ ^)^.

Os antioxidantes naturais reduzem os efeitos adversos dos radicais livres por possuírem a capacidade de captar e neutralizar as espécies reativas de oxigênio (ERO), impedindo a peroxidação lipídica. Essa neutralização é essencial, especialmente em situações de aumento do estresse oxidativo, como ocorre na DMG^(^ ^3^ ^)^. Dentre essas substâncias, destaca-se a vitamina E, micronutriente essencial, que corresponde a um grupo de oito compostos lipossolúveis classificados em α-, β-, γ- e δ-tocoferol ou tocotrienol, sendo o α-tocoferol o biologicamente mais ativo^(^ ^4^ ^)^. 

Sabe-se que a transferência placentária da vitamina E durante a gestação é limitada e que as reservas formadas desse micronutriente no recém-nascido são baixas, tornando o aleitamento materno exclusivo a única fonte de aquisição para suprir suas necessidades nutricionais^(^ ^4^ ^)^. Essa oferta é extremamente importante, pois a mudança para um ambiente hiperoxêmico no momento do parto eleva o risco de formação de radicais livres^(^ ^5^ ^)^.Pesquisadores avaliaram a relação entre vitamina E e diabetes e verificaram que a suplementação desta relaciona-se à produção de insulina e à proteção das células-beta do pâncreas, revelando que a vitamina E pode se relacionar diretamente ao diabetes^(^ ^6^ ^)^. 

Diante da importância da vitamina E para o binômio mãe-filho, este trabalho teve o objetivo de verificar a concentração de α-tocoferol no leite colostro e no soro de mães diabéticas e saudáveis, bem como comparar os valores vitamínicos entre os grupos estudados. A relevância deste trabalho consiste em verificar se a DMG pode ser um fator de risco para deficiência em vitamina E na parturiente e/ou no recém-nascido, considerando-se que o leite materno é a única fonte dessa vitamina para recém-nascidos em aleitamento materno exclusivo. 

## Método

O estudo, do tipo transversal, foi realizado com 51 parturientes voluntárias, sendo 20 diabéticas e 31 sem nenhuma doença associada, todas atendidas em maternidade pública de Natal, Rio Grande do Norte. Coletaram-se as amostras de outubro de 2011 a agosto de 2012.

Para o cálculo amostral, utilizou-se o *software* G*Power, versão 3.1.7^(^ ^7^ ^)^. O cálculo foi realizado considerando-se os seguintes parâmetros: valor de α=5%, poder do teste de 80% e expectativa da medida de efeito de 0,81. O tamanho total indicado para a amostra foi de 50 casos.

Os critérios de exclusão para o grupo de parturientes saudáveis foram: presença de doenças (diabetes, hipertensão, neoplasias, doenças do trato gastrintestinal e hepáticas, cardiopatias, infecciosas, sífilis e HIV positivo); utilização de suplementos vitamínicos contendo vitamina E durante a gestação; conceptos múltiplos ou com malformação. Quanto às parturientes com diabetes gestacional, excluíram-se as insulinodependentes, com conceptos múltiplos ou com malformação e aquelas que fizeram uso de suplementos vitamínicos durante a gestação. O diagnóstico de DMG foi realizado pela equipe médica durante o pré-natal das participantes. Para rastreamento positivo durante o pré-natal, independentemente do período gestacional, estabeleceu-se que a glicemia de jejum materna deveria ser igual ou superior a 85mg/dL. Para o diagnóstico do DMG, utilizaram-se os pontos de corte de 110mg/dL para a glicemia de jejum e de 140mg/dL para o valor do Teste Oral de Tolerância à Glicose (TOTG), realizado duas horas após sobrecarga oral com 75g de glicose. 

Todos os procedimentos realizados no estudo foram aprovados pelo Comitê de Ética em Pesquisa do Hospital Universitário Onofre Lopes da Universidade Federal do Rio Grande do Norte (UFRN). Todas as participantes foram esclarecidas sobre a pesquisa e assinaram o termo de consentimento livre e esclarecido. 

Os dados sobre as características maternas e obtétricas foram obtidos do prontuário da parturiente, do cartão de acompanhamento pré-natal e por meio de entrevistas realizadas pelos pesquisadores. O estado nutricional antropométrico pré-gestacional foi determinado pelo índice de massa corpórea (IMC), calculado pela razão entre o peso corpóreo habitual da mulher antes da gestação e sua estatura elevada ao quadrado. As mulheres com IMC <19,8kg/m^2^ foram classificadas como baixo peso; aquelas com IMC entre 19,8-26kg/m^2^ como eutrofia; as com IMC entre 26-29kg/m^2^ como portadoras de sobrepeso e as mulheres com IMC>29kg/m^2^ foram classificadas como obesas^(^ ^8^ ^)^. 

O ganho de peso gestacional das participantes foi calculado pela diferença entre o peso corpóreo pré-parto e o peso pré-gestacional e avaliado de acordo com o preconizado pelo Instituto de Medicina^(^ ^8^ ^)^. As mulheres com baixo peso pré-gestacional deveriam ganhar entre 12,5-18kg até o final da gestação; aquelas com peso adequado pré-gestacional, entre 11,5-16kg; as com sobrepeso, entre 7-11,5kg; e as mulheres com obesidade deveriam ganhar aproximadamente 7kg até o final da gestação.

Os dados sobre as características dos recém-nascidos das mães participantes foram obtidos do prontuário médico. Classificaram-se os recém-nascidos segundo a idade gestacional em pré-termo (os que nasceram com menos de 37 semanas) e a termo (os que nasceram entre a 37^a^ e 42^a ^semana)^(^ ^9^ ^)^. Os recém-nascidos também foram classificados segundo o peso ao nascer em baixo peso (peso inferior a 2500g); adequado (peso entre 2500 e 4000g); e macrossômicos (peso acima de 4000g)^(^ ^10^ ^)^. 

No primeiro dia pós-parto, coletaram-se 2mL de colostro e 5mL de sangue de cada parturiente envolvida no estudo. Essas amostras biológicas foram colhidas de manhã, após jejum de 8 a 12 horas. Obteve-se o colostro por expressão manual de uma única mama, para evitar flutuação no teor de gordura, e obteve-se o sangue por punção venosa. As amostras foram coletadas em tubos de polipropileno protegidos da luz e transportados em caixas térmicas ao Laboratório de Bioquímica dos Alimentos e da Nutrição do Departamento de Bioquímica da UFRN. As amostras de colostro foram submetidas a banho-maria a 37°C por cinco minutos e agitadas para homogeneização da amostra. Em seguida, retirou-se uma alíquota de 500µL, colocada em tubo de polipropileno protegido da luz e armazenada a -18°C até o momento da análise. O sangue foi centrifugado para a retirada do soro, separando-se 1mL e armazenando-o da mesma forma que o colostro.

A concentração de alfa-tocoferol nas amostras de soro e de colostro foi determinada por cromatografia líquida de alta eficiência (CLAE). A análise bioquímica do α-tocoferol nas amostras biológicas ocorreu conforme a adaptação do método de extração utilizado por Ortega et al^(^ ^11^ ^)^, como descrito adiante. Para 1mL de soro, utilizou-se 1mL de etanol 95% (Merck^(r)^, USA); em seguida, houve duas extrações com 2mL de hexano (Merck^(r)^, USA) e evaporação de 2mL de extrato hexânico em banho-maria a 37ºC. Para 500µL de colostro, adicionaram-se 500µL de etanol 95% (Merck^(r)^, USA); em seguida, houve duas extrações com 2mL de hexano (Merck^(r)^, USA) e evaporação nas mesmas condições do soro. Após esses procedimentos, as amostras foram redissolvidas em 500µL de etanol absoluto (Vetec^(r)^, Brasil) e 20µL foram aplicados no aparelho CLAE (modelo LC-10 AD, Shimadzu Corporation^(r)^, Japão) acoplado a um detector UV-VIS (SPD-10 A, Shimadzu Corporation^(r)^, Japão) e a um integrador Chromatopac C-R6A (Shimadzu Corporation^(r)^, Japão). Utilizou-se coluna de fase reversa (CLC-ODS (M), Shim-pack, Japão) de 4,6mm d.i.x25cm de comprimento. A fase móvel utilizada nessas análises foi metanol 100% (J. T. Baker, México) em um fluxo de 1,0mL/minuto. 

Identificou-se o α-tocoferol nas amostras por comparação entre o tempo de retenção dos picos obtidos nos cromatogramas e aquele obtido com a aplicação do padrão de α-tocoferol Sigma^(r)^. A fim de quantificar o α-tocoferol nas amostras biológicas, utilizou-se uma curva padrão para padronização externa. A concentração do padrão foi confirmada pelo coeficiente de extinção específico em etanol absoluto^(^ ^12^ ^)^, ε 1%, 1cm=75,8 a 292nm (Sigma^(r)^, USA). 

Concentrações de α-tocoferol sérico materno acima de 697,7µg/dL são consideradas aceitáveis, conforme os valores estabelecidos por Sauberlich^(^ ^13^ ^)^.

Para determinar a precisão e a exatidão, realizaram-se os testes de recuperação e de repetitividade expressa por desvio padrão relativo (DPR), com três concentrações, (50, 100 e 200ng/mL), contemplando-se a faixa de variação dentro da curva de linearidade. Determinou-se também o limite de detecção (LD) e de quantificação (LQ).

O α-tocoferol foi detectado no colostro e no soro. A taxa de recuperação média de α-tocoferol para o soro foi de 100% e, para o colostro, de 98,8%. As medições caracterizaram-se por repetibilidade satisfatória, com DPR de soro e colostro de 7,7 e 4,2%, respectivamente. 

Para se determinar o LD e o LQ, diluíram-se as amostras de soro e de colostro, cujas concentrações eram conhecidas. A cada diluição, as amostras foram aplicadas e o pico observado, de forma que o LD foi determinado quando não houve mais distinção entre o ruído e o sinal analítico, sendo atingido na concentração de 3,18ng/mL. O LQ foi determinado quando se detectou o sinal analítico na menor diluição, sendo equivalente a 6,36ng/mL, conforme o método visual^(^ ^14^ ^)^. A curva de calibração foi realizada com soluções padrão de α-tocoferol (Sigma^(r))^. A curva de calibração para α-tocoferol foi linear (r^2^=0,9998) e obtida dentro de 1,2-41,3µg/mL. 

Analisaram-se os dados pelo *Statistic Release *7^(r)^, sendo apresentados em média e desvio padrão. Utilizou-se o teste de Kolmogorov-Smirnov para verificar a normalidade das variáveis métricas de interesse. Após a comprovação da normalidade, aplicou-se o teste *t* de Student para variáveis independentes, a fim de averiguar se havia diferença significante entre as médias de α-tocoferol dos grupos estudados. Utilizou-se a correlação de Pearson para avaliar a relação entre a concentração de α-tocoferol no soro e no colostro, sendo significante *p*<0,05. Essa correlação foi realizada para cada grupo estudado.

## Resultados

As parturientes que participaram deste estudo foram distribuídas para análise dos resultados em dois grupos: Controle (n=31) e Grupo de Diabetes Melito Gestacional (n=20). 

As parturientes incluídas no Grupo Controle eram, em sua maioria, adultas (81%), foram submetidas ao parto vaginal (55%) e tiveram filhos a termo (96%) e com peso adequado (87%). Quanto ao peso pré-gestacional, 42% foram classificadas como eutróficas. A média da idade gestacional (IG) dos recém-nascidos das parturientes desse grupo foi de 39±1,2 semanas e somente um recém-nascido foi classificado como prematuro, com IG de 36 semanas. Quanto às parturientes incluídas no grupo das diabéticas, todas eram adultas e a maioria teve a cesariana (90%) como via de parto e filhos a termo (71%). Quanto à classificação do peso pré-gestacional, 44% eram obesas. Quanto ao peso dos recém-nascidos desse grupo, 33,3% nasceram com peso insuficiente, 33,3% com peso adequado e 33,3% com macrossomia. A média da IG dos recém-nascidos das mães incluídas nesse grupo foi de 37±2 semanas e quatro recém-nascidos foram classificados como prematuros, sendo a idade gestacional média de 35±1 semanas (Tabela 1). 

**Tabela 1:** Caracterização da população estudada com base em características maternas, obstétricas e do neonato

Nas parturientes do Grupo Controle, a concentração média de α-tocoferol encontrada no soro foi de 1.059,5±372,7µg/dL (mínimo: 482,3; máximo: 2.405,8). Para as mulheres do grupo das diabéticas, a média foi de 1.391,4±531,5µg/dL (mínimo: 368,0; máximo: 2.311,5), havendo diferença significante entre os grupos (*p*<0,01) (Figura 1). A concentração de α-tocoferol no colostro foi de 1.483,1±533,8µg/dL (mínimo: 645,5; máximo: 2.360,1) para as participantes do Grupo Controle e de 1.368,8±681,8µg/dL (mínimo: 505,6; máximo: 2.750,6) para as diabéticas, não havendo diferença entre os grupos (Figura 2). Não se verificou correlação entre o soro e o colostro tanto para o Grupo Controle (*p*>0,05; r=0,12) quanto para o Grupo de Diabetes Melito Gestacional (*p*>0,05; r=-0,11). A correlação para ambos os grupos está expressa nas Figuras 3 e 4. 

**Figura 1:** Concentração de a-tocoferol no soro de lactantes do Grupo Controle e do Grupo de Diabetes Melito Gestacional

**Figura 2:** Concentração de a-tocoferol no colostro de lactantes do Grupo Controle e do Grupo de Diabetes Melito Gestacional

**Figura 3:** Correlação entre a concentração de a-tocoferol no soro e no colostro de lactantes do Grupo Controle

**Figura 4:** Correlação entre a concentração de a-tocoferol no soro e no colostro de lactantes do Grupo de Diabetes Melito Gestacional

## Discussão

O estado nutricional materno pré-gestacional e durante a gravidez é um dos fatores modificáveis mais importantes para a saúde da gestante e de seu bebê. A inadequação do estado nutricional materno influencia o resultado da gravidez e as condições de crescimento apresentadas pelo recém-nascido, pois o período gestacional é uma fase em que as necessidades nutricionais estão elevadas, decorrentes dos ajustes ﬁsiológicos da gestante e das demandas de nutrientes para o crescimento fetal^(^ ^15^ ^)^.

A partir das médias das concentrações séricas de α-tocoferol observadas em ambos os grupos, o estado nutricional bioquímico do soro é aceitável^(^ ^13^ ^)^, não havendo deficiência relacionada ao diabetes. Quanto ao colostro, verificou-se que tanto o Grupo Controle quanto o Grupo de Diabetes Melito Gestacional atendem ao requerimento nutricional do bebê, considerando-se que o volume estimado da ingestão do colostro é de 500mL/dia^(^ ^16^ ^)^, sendo o resultado comparado diretamente com o valor de referência do *Institute of Medicine* do ano 2000^(17) ^para a ingestão do nutriente (4mg/dia). 

A partir dos resultados, observou-se que o soro das mulheres com DMG apresentou maior concentração de α-tocoferol do que o de mães saudáveis. Porém, Grissa *et al**^(^* ^18^ ^)^ e Peuchant *et al**^(^* ^19^ ^)^, em estudo realizado com gestantes, relataram um decréscimo das concentrações de vitamina E no soro de mães diabéticas, quando comparado ao soro das mães saudáveis. Suhail *et al**^(^* ^20^ ^)^ também verificaram decréscimo dessa concentração sérica em relação ao grupo controle. Entretanto, tais autores realizaram a coleta no momento do parto, diferentemente deste estudo, em que se realizaram as coletas das amostras no primeiro dia pós-parto. 

Em estudos realizados por Lammi-Keefe *et al**^(^* ^21^ ^)^ e Dey *et al**^(^* ^22^ ^)^, não houve diferença significante entre a quantidade de tocoferol no soro do grupo diabético e do grupo saudável. O estudo de Lammi-Keefe *et al**^(^* ^21^ ^)^ foi o primeiro realizado com lactantes com diabetes melito insulinodependentes e seu respectivo grupo controle, composto por lactantes sem nenhum tipo de doença. O segundo estudo foi realizado na Índia e analisou o tocoferol em gestantes diagnosticadas com diabetes gestacional. Entretanto, outro estudo realizado na Índia por Santra *et al**^(^* ^23^ ^)^ encontrou maior quantidade de vitamina E no soro de mulheres com DMG, em comparação ao grupo controle, corroborando o resultado obtido nesta pesquisa. A quantidade de vitamina E sofreu ainda aumento gradual após quatro semanas, mediante nova coleta.

 A concentração sérica aumentada de α-tocoferol pode estar relacionada às alterações metabólicas que ocorrem no organismo materno devido ao DMG. No final da gestação, pressupõe-se redução na secreção de insulina, levando a uma resposta hormonal que faz os tecidos dependentes de insulina metabolizarem os lipídeos em vez dos carboidratos, iniciando um processo de lipólise^(^ ^24^ ^-^ ^26^ ^)^, que culmina em maior liberação de ácidos graxos livres na circulação^(^ ^27^ ^)^. Uma vez que o principal meio de estocagem de α-tocoferol no organismo é o tecido adiposo^(^ ^28^ ^)^, essa vitamina pode sofrer influência do aumento da lipólise, fazendo com que gestantes diabéticas apresentem concentrações elevadas de tocoferol sérico. 

Há evidências de que o aumento do estresse oxidativo pode aumentar a atividade de antioxidantes enzimáticos em animais, como uma condição adaptativa do organismo. De Angelis *et al* verificaram que ratos diabéticos tiveram aumento de 92% na atividade da catalase e de 27% da glutationa S-transferase em músculos, em comparação ao grupo controle. Conforme a vitamina E atua como antioxidante, é oxidada e transformada em radical livre (tocoferil), necessitando, assim, de um sistema de regeneração que permita a recuperação de sua função antioxidante. Para isso, outros antioxidantes atuam removendo o radical livre da molécula do tocoferol, como o ácido ascórbico, a enzima glutationa-reduzida e a Coenzima Q10^(^ ^29^ ^,^ ^30^ ^)^. Giannubilo *et al**^(^* ^31^ ^)^ verificaram concentração plasmática significativamente maior da coenzima Q10 no final da gestação (36-40 semanas) em pacientes com DMG, quando comparadas ao grupo controle. Para explicar essa diferença, os autores sugerem haver um mecanismo compensatório em resposta ao estresse oxidativo associado à hiperglicemia e à resistência à insulina em pacientes com DMG. 

Diante dos estudos expostos e dos resultados obtidos, apesar de poucos artigos publicados, pode-se levantar a hipótese de que o aumento do α-tocoferol no sangue de mães com DMG se relacione à tentativa de reagir à doença e minimizar os danos causados, na forma de mecanismo de compensação, observado tanto no aumento da lipólise diante da baixa disponibilidade de energia quanto no aumento da atividade antioxidante diante do estresse oxidativo.

Quanto à concentração de α-tocoferol no colostro de mães diabéticas, encontraram-se poucos estudos a esse respeito. Apesar da escassez de trabalhos, Lammi-Keefe *et al**^(^* ^21^ ^)^ analisaram a quantidade de vitamina E no leite de diabéticas no sétimo, 14º, 42º e 84º dias de lactação. Em seus resultados, observa-se que a quantidade de tocoferol no colostro, que corresponde à amostragem do sétimo dia, foi maior em comparação ao grupo das mães saudáveis, sendo essa diferença significante. No mesmo grupo, a concentração vitamínica mostrou-se abaixo do valor de referência adotado pelos autores. Nesse mesmo estudo, também se verificou que as concentrações de α-tocoferol para as mães diabéticas diferem das encontradas para o grupo controle, tanto no colostro quanto no soro; porém, em ambos os grupos, não houve correlação significativa entre eles, resultado semelhante ao de pesquisas anteriores, como as de Azeredo e Trugo^(^ ^32^ ^)^, Dimenstein *et al**^(^* ^33^ ^)^ e Lira *et al**^(^* ^34^ ^)^. Isso reforça a hipótese de que há um mecanismo diferente de transferência do tocoferol para a glândula mamária a fim de suprir a necessidade do recém-nascido, não havendo dependência quanto à situação sérica materna desse nutriente^(^ ^32^ ^)^.

Nesse contexto, é essencial a existência desse mecanismo independente, pois o organismo materno deve garantir a transferência da vitamina E para o leite em quantidades suficientes para a formação das reservas do recém-nascido, garantindo a proteção do organismo contra a toxicidade do oxigênio e estimulando o desenvolvimento e a manutenção do sistema imune^(^ ^4^ ^)^. Além de sua ação como antioxidante ser de extrema importância para o recém-nascido, a vitamina E também desempenha função moduladora na pós-tradução e na transcrição de genes, ação anti-inflamatória e de estabilidade das membranas^(^ ^5^ ^,^ ^35^ ^,^ ^36^ ^)^.

Tanto as parturientes saudáveis quanto aquelas com DMG apresentaram estado nutricional adequado quanto à vitamina E. Apesar disso, a concentração de α-tocoferol sérico foi significativamente maior no grupo diabético, sendo um fator de proteção contra o estresse oxidativo para essas mulheres. Sugere-se que esse aumento decorra de alterações metabólicas associadas ao DMG, como a lipólise.

Quanto às limitações do estudo, vale lembrar que o poder do teste expressa a probabilidade de se detectar um efeito verdadeiro^(^ ^37^ ^)^. A probabilidade de um pesquisador cometer um erro do tipo II, em que ele não rejeita a hipótese nula quando ela é falsa para a população, designa-se por erro beta (β)^(^ ^38^ ^)^. Para o cálculo amostral deste estudo, utilizou-se poder do teste de 80% e valor do erro beta de 20%. Além disso, incluíram-se algumas mães de recém-nascidos prematuros. Entretanto, alguns trabalhos evidenciam que a idade gestacional não possui influência sobre os níveis de α-tocoferol no colostro^(^ ^39^ ^-^ ^41^ ^)^. Finalmente, destaca-se a falta de avaliação dietética das parturientes incluídas no estudo e o fato de a coleta de leite materno ter sido realizada somente em um período da lactação. De qualquer modo, trabalhos como este, que verificam a associação entre doenças e a concentração de vitaminas no soro e no leite materno, são relevantes para se definirem grupos de riscos para deficiência vitamínica. 

O DMG não se associou com a concentração de α-tocoferol no colostro e não parece ser um fator de risco para a deficiência de vitamina E em recém-nascidos cujas mães possuem essa doença. As concentrações vitamínicas em ambos os grupos suprem o requerimento diário de vitamina E para bebês. Além disso, as parturientes com DMG possuíam maiores concentrações de α-tocoferol no soro, o que pode ser um reflexo de alterações fisiológicas e bioquímicas que caracterizam a doença. Novos estudos devem ser realizados a fim de se verificar a influência de outras doenças comuns na gestação sobre as concentrações de α-tocoferol e outras vitaminas no soro e no leite maternos.
